# Clinical pathological analysis of primary small cell neuroendocrine carcinoma of the cervix: A retrospective study

**DOI:** 10.1097/MD.0000000000046168

**Published:** 2025-11-21

**Authors:** Pu Jin, Congyang Gu, Shiyue Huang, Xiaohua Liu

**Affiliations:** a Department of Pathology, Xindu District People’s Hospital of Chengdu, Chengdu, Sichuan, China; b Department of Pathology, The First People’s Hospital of Neijiang, Neijiang, Sichuan, China.

**Keywords:** cervix, differential diagnosis, HPV, immunohistochemistry, missed diagnosis, neuroendocrine carcinoma, small cell carcinoma

## Abstract

To explore the clinical and pathological features of primary small cell neuroendocrine carcinoma of the cervix. Clinical data of 4 patients with primary small cell neuroendocrine carcinoma of the cervix were collected, and the clinical features, histopathological characteristics, immunohistochemical staining results, treatment, and prognosis were summarized and analyzed. The relevant literature was reviewed and summarized. The age of the 4 patients ranged from 48 to 62 years, with an average age of 54.3 years. Patients 1, 2, and 3 sought medical attention because of irregular vaginal bleeding, whereas patient 4 presented with lower abdominal pain and discomfort. Histologically, all 4 cases were small-cell neuroendocrine carcinomas. Immunophenotyping: tumor cells were diffusely strongly positive for P16, and expressed 2 or more neuroendocrine markers, with positive rates of CD56 100% (4/4), synaptophysin (SYN) 100% (4/4), and chromogranin (CgA) 50% (2/4). The first and second cases were positive for TTF-1, epithelial marker panCK was positive, and Ki-67 proliferation index was >70% in all cases. This is a relatively rare and highly malignant cervical tumor with strong invasiveness and a poor prognosis. This may be related to human papillomavirus infection. A definitive diagnosis can be made based on the histopathological morphology and immunophenotype.

## 1. Introduction

Cervical neuroendocrine carcinoma is an extremely rare pathological type of cervical cancer, accounting for only 1% to 3% of all cervical cancers, with small cell neuroendocrine carcinoma (SCNEC) being the most common.^[1]^ The limited biopsy tissue and the observed cellular morphology under a microscope make it prone to misdiagnosis. This study retrospectively analyzed the clinical and pathological characteristics of 4 cases of SCNEC to improve our understanding of this tumor.

## 2. Materials and methods

### 2.1. General information

Patients with SCNEC were recruited from January 2018 to January 2024 at the Department of Pathology, People’s Hospital of Xindu District, Chengdu, and the Department of Pathology, The First People’s Hospital of Neijiang City, for a total of 4 cases. Case 1 was a cervical conization specimen, Case 3 was a surgically resected specimen, and the remaining 2 cases were cervical biopsy specimens. Clinical data were collected and pathological slides were reviewed. The diagnosis of cervical SCNEC was based on the pathological diagnostic criteria of the 2020 WHO Classification of Tumors of the Female Reproductive System.

### 2.2. Methods

All specimens were fixed with 10% neutral buffered formalin, paraffin-embedded, and sectioned at a thickness of ~4 μm, followed by hematoxylin-eosin staining. Immunohistochemical staining was performed using the (polymer) 2-step method, with 3,3'-Diaminobenzidine as the chromogen and hematoxylin for counterstaining. The primary antibodies used, including panCK, Syn, CgA, CD56, p16, p63, p40, LCA, p53, TTF-1, and Ki-67, were purchased from Henan Sinotech Biotechnology Co., Ltd., and the secondary antibody kits were obtained from Leica. The diagnoses of all 4 cases were confirmed by 2 or more senior pathologists.

## 3. Results

### 3.1. Clinical features

Case 1: Irregular vaginal bleeding for over 5 months, a tongue-like mass was observed at the cervical os with contact bleeding; human papillomavirus (HPV) type 18 was positive. Color Doppler Ultrasound: The uterus is retroverted, with the corpus uteri measuring ~2.8 cm × 3.0 cm × 3.0 cm, heterogeneous myometrial echo. A hypoechoic nodule, measuring ~0.7 cm × 0.5 cm, is seen in the posterior wall of the uterus, clear boundaries. No obvious space-occupying lesions seen in the bilateral adnexal regions. Pelvic cavity: No obvious free anechoic area was seen in the uterine rectal fossa (Table 1). Case 2: Irregular vaginal bleeding for over 4 months, physical examination revealed the disappearance of the cervix and vaginal fornix, presence of an irregular cauliflower-like neoplasm measuring 2 to 3 cm, extensive necrotic tissue, a strong foul odor, and active bleeding upon touch. The uterus and bilateral adnexa were not palpable, but a hard, large mass was palpated in the abdomen, with a diameter of about 15 cm. Pelvic Magnetic Resonance Imaging: Cervix, corpus uteri, upper middle segment of the anterior vaginal wall, and irregular large soft tissue mass in the pelvis. The possibility of neoplastic changes is high, considering cervical cancer. The lesion involves the trigone of the bladder, the posterior wall, the lower segment of the left ureter, and the parametrium. The size of the lesion is ~12.2 cm × 10.6 cm × 10.1 cm (Table 1). Case 3: Irregular vaginal bleeding for 20 days, physical examination shows a smooth cervical surface, which feels hard upon touch; ultrasound reveals an abnormal cervical shape with an anteroposterior diameter of about 3.3 cm, several anechoic nodules are observed internally, the largest measuring ~1.2 cm × 0.8 cm, and a hypoechoic mass is found within the cervix, measuring about 1.8 cm × 1.6 cm × 1.3 cm; HPV type 16 is positive (Table 1). Case 4: Menopausal for 1 year, with lower abdominal discomfort and pain; upon examination, the cervix shows significant contact bleeding and a cauliflower-like neoplasm is observed. Ultrasonography: The cervix was normal with no significant abnormal echo. HPV type 16 is positive (Table 1).

**Table 1:** Clinical and pathological data of 4 patients with small cell neuroendocrine carcinoma (SCNEC).

Serial number	Age (yr)	Clial symptoms	Gross manifestations	HPV	Microscopic features	Pathological diagnosis	Lymph node metastasis	Treatment	Follow-up
1	62	Irregular vaginal bleeding for over 5 mo	Tongue-shaped polyp, diameter 1.2 cm	Type 18 +	Nuclei are deeply stained, cytoplasm is scant, and the nuclear-to-cytoplasmic ratio is high	Small cell neuroendocrine carcinoma	Unclear	Total hysterectomy with bilateral salpingo-oophorectomy and pelvic lymphadenectomy	Follow-up for 13 mo without recurrence
2	51	Irregular vaginal bleeding for over 4 mo	Cauliflower-like neoplasm, diameter 2–3 cm	Not examined	Tumor cells are diffusely and patchily distributed with significant necrosis	Small cell neuroendocrine carcinoma	Unclear	Total hysterectomy with bilateral salpingo-oophorectomy and pelvic lymphadenectomy	Death
3	48	Irregular vaginal bleeding for 20 d	“Cylindrical” cervix, diameter 4.5 cm	Type 16 +	Tumor diffusely infiltrates the cervical stroma without invasion of the cervical epithelium	Small cell neuroendocrine carcinoma	Left pelvic lymph node metastasis (2/14)	Total hysterectomy with bilateral salpingo-oophorectomy and pelvic lymphadenectomy	Death
4	56	Menopause for 1 yr, with lower abdominal pain and discomfort	Cauliflower-like neoplasm	Type 16 +	Nuclei are deeply stained, with a pronounced “crowding” phenomenon	Small cell neuroendocrine carcinoma	None	Radiotherapy plus chemotherapy	Follow-up for 23 mo without recurrence

### 3.2. Gross examination

Case 1: Multiple pieces of grayish-white, pale brown, irregularly shaped tissue, with a total volume of 1.5 cm × 1.2 cm × 0.2 cm. Case 2: 5 small pieces of grayish-white, pale red tissue, with a total volume of 1.3 cm × 1.1 cm × 0.5 cm. Case 3: The cervix is hard and has a “barrel-like” shape, with a smooth surface; the volume of the hard area is 4.5 cm × 2.5 cm × 1.3 cm. Case 4: A pile of grayish-white fragmented tissue, with a total volume of 2.0 cm × 2.0 cm × 0.5 cm.

### 3.3. Under light microscopy observation

**Figure 1.**
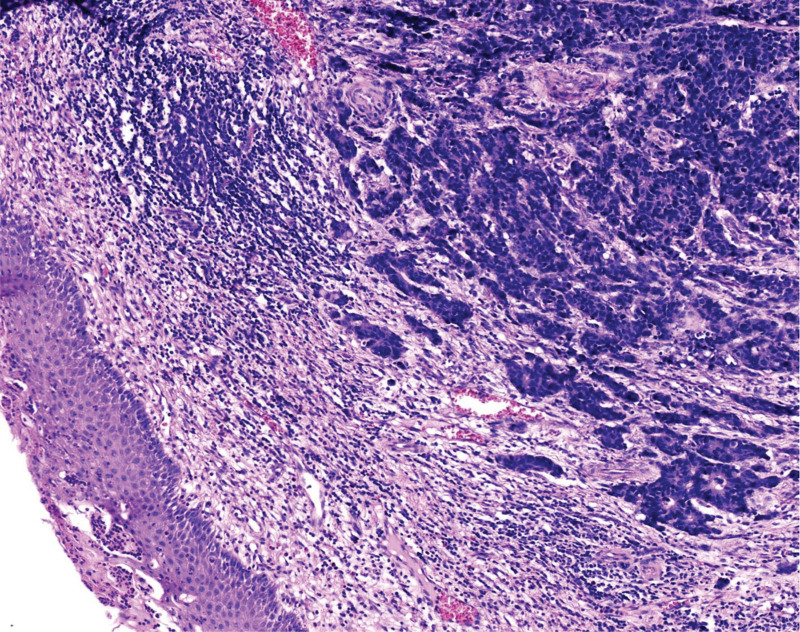
The tumor tissue diffusely infiltrates the cervical stroma without invading the cervical surface epithelium. HE staining, ×100.

**Figure 2.**
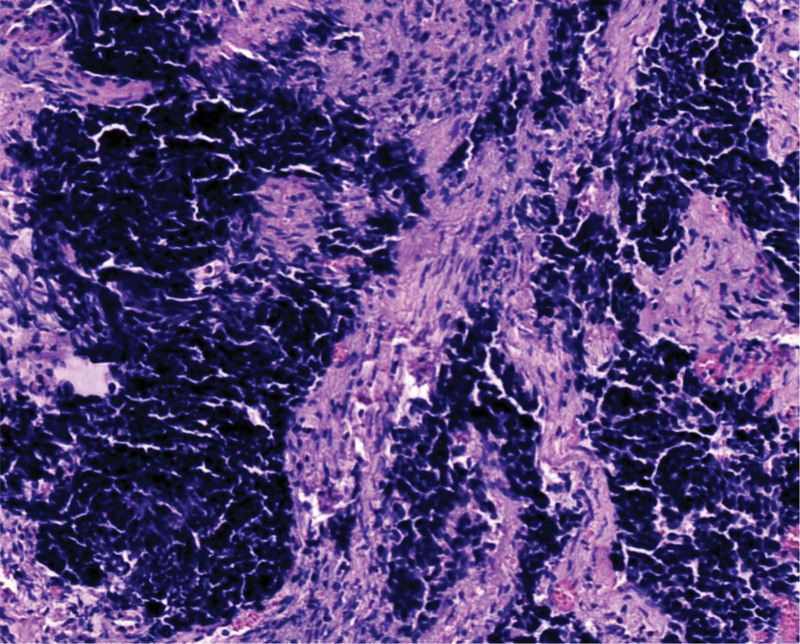
The tumor cells have scant cytoplasm, deeply stained nuclei, and exhibit a “squeezing” phenomenon. HE staining, ×200.

**Figure 3.**
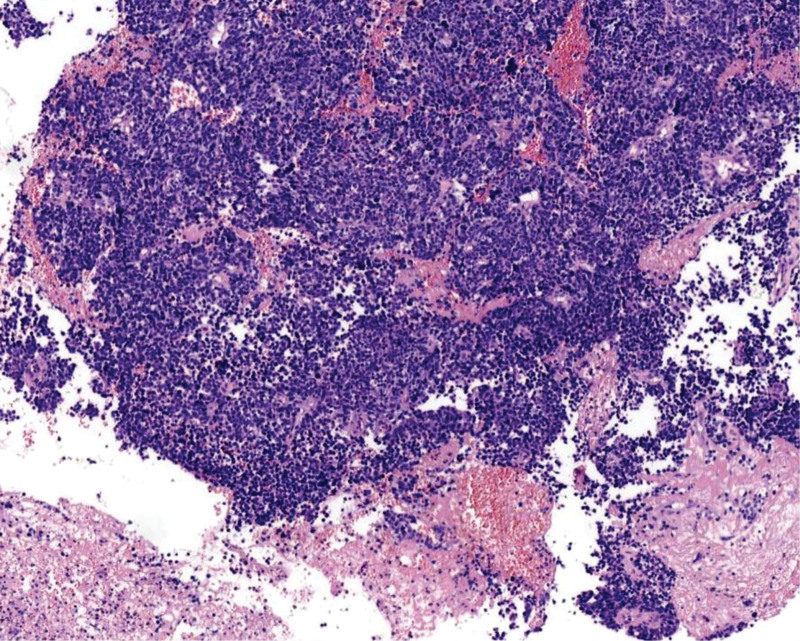
The tumor cells are diffusely arranged in sheets, with areas of necrosis visible in some regions. HE staining, ×100.

The tumor exhibited infiltrative growth with cells arranged in sheets, nests, and cords. The tumor tissue diffusely infiltrated the cervical stroma, without invading the cervical surface epithelium (Fig. 1). The tumor cells are oval or spindle-shaped with significant atypia, indistinct cell boundaries, and a prominent “squeezing” phenomenon. The cell nuclei are oval, spindle-shaped, oat-shaped, deeply stained, with fine chromatin, scant cytoplasm, a high nuclear-to-cytoplasmic ratio, indistinct nucleoli, and easily visible mitotic figures (Fig. 2). Necrosis was also observed in some areas (Fig. 3).

### 3.4. Immunophenotype

**Figure 4.**
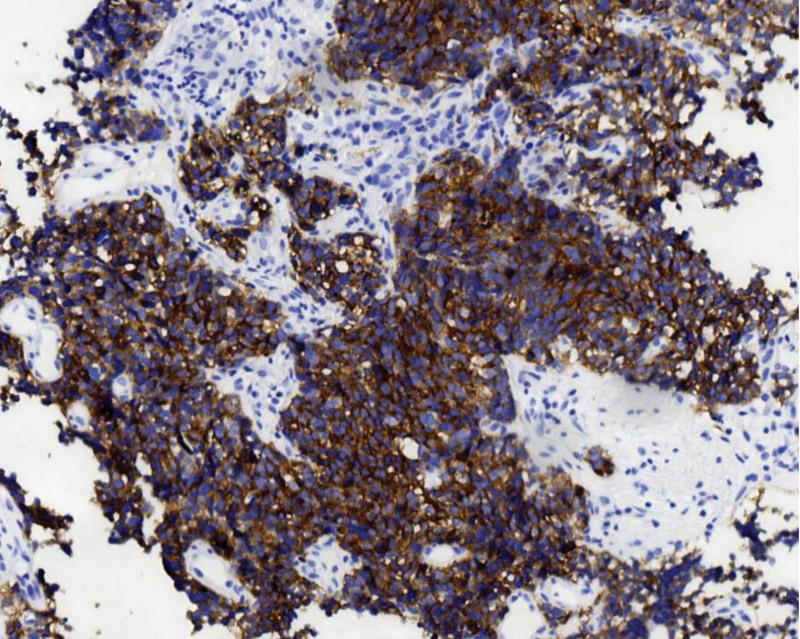
Syn is diffusely expressed. Polymer 2-step method. Immunohistochemical staining, ×200.

**Figure 5.**
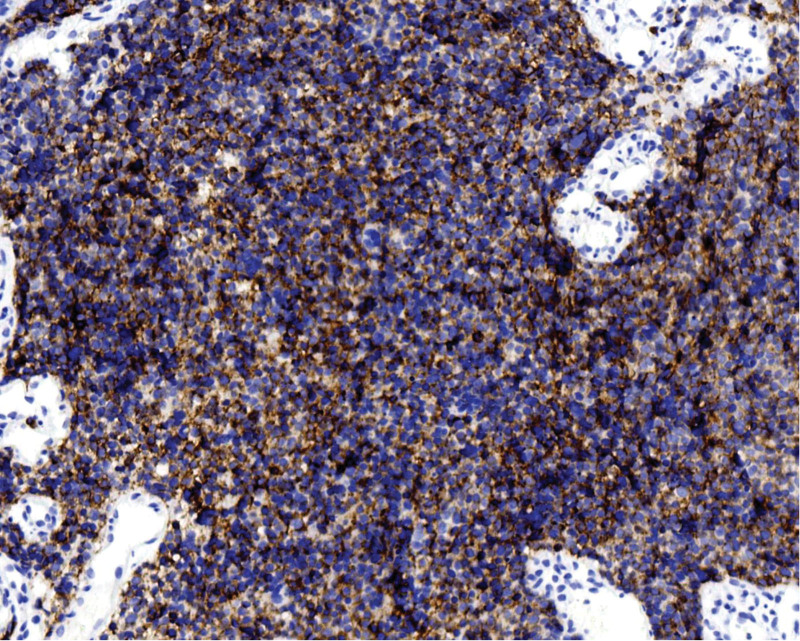
CD56 is diffusely expressed. Polymer 2-step method. Immunohistochemical staining, ×200.

**Figure 6.**
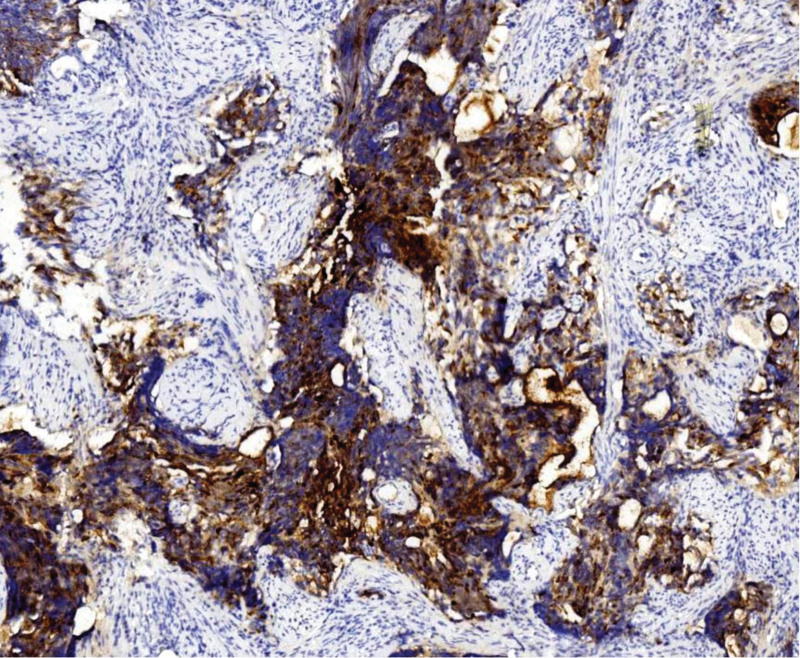
CgA is diffusely expressed. Polymer 2-step method. Immunohistochemical staining, ×100.

**Figure 7.**
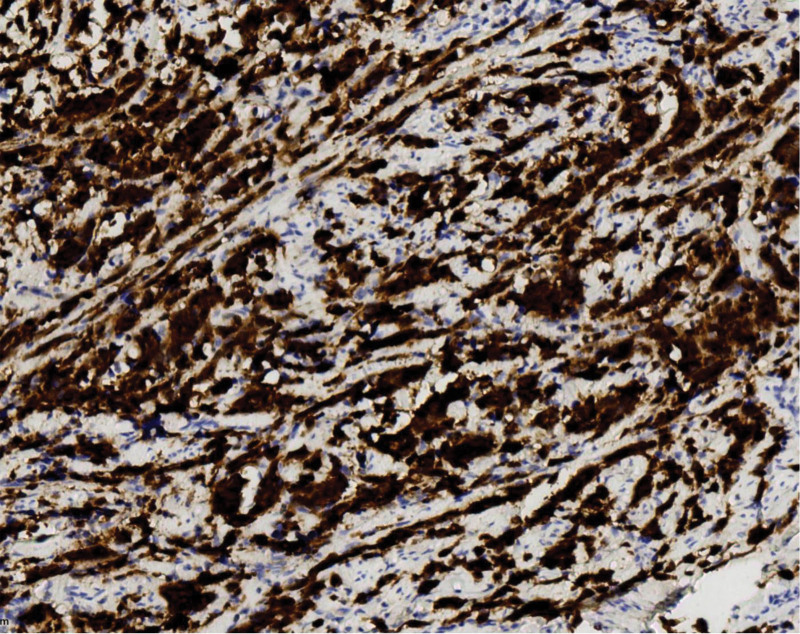
P16 is diffusely expressed. Polymer 2-step method. Immunohistochemical staining, ×200.

**Figure 8.**
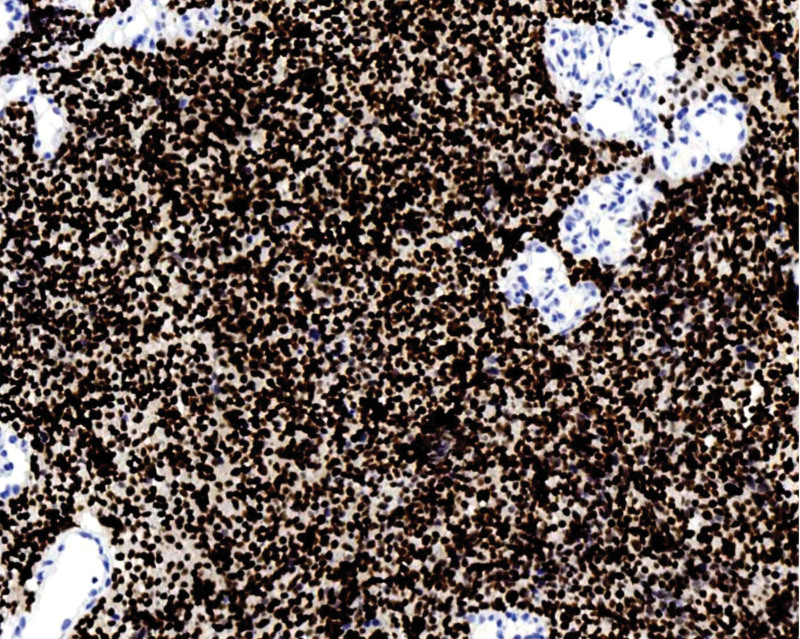
TTF-1 is diffusely expressed. Polymer 2-step method. Immunohistochemical staining, ×200.

**Figure 9.**
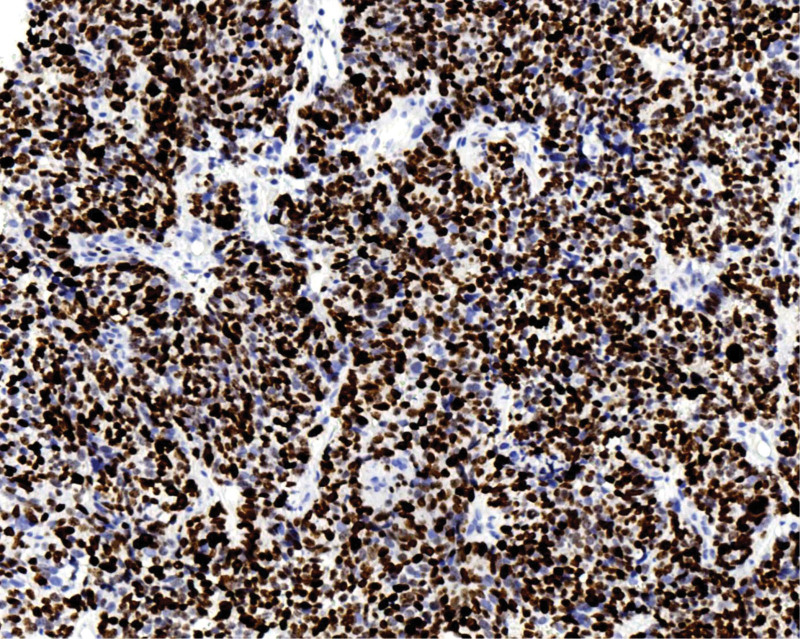
Ki-67 shows ~90% positive expression. Polymer 2-step method.Immunohistochemical staining, ×200.

Tumor cells expressed 2 or more neuroendocrine markers: synaptophysin (SYN) 100 % (4/4) (Fig. 4), CD56 100 % (4/4) (Fig. 5), and chromogranin A (CgA) 50 % (2/4) (Fig. 6). The epithelial marker panCK was positive, P16 showed diffuse strong positivity in all cases (Fig. 7), cases 1 and 2 were TTF-1 positive (Fig. 8), and the Ki-67 proliferation index was >70 % in every case (Fig. 9, Table 2).

**Table 2:** Immunohistochemical results of 4 cases of small cell neuroendocrine carcinoma (SCNEC) patients.

Numbering	Syn	CD56	CgA	panCK	p16	p63	p40	LCA	p53	TTF-1	Ki67
1	+	Portion+	+	+	Diffuse strong positivity	−	−	−	Wild type	+	70%+
2	+	+	−	+	Diffuse strong positivity	−	−	−	Wild type	+	90%+
3	Focal+	Portion+	+	+	Diffuse strong positivity	−	−	−	Wild type	−	80%+
4	+	+	−	+	Diffuse strong positivity	−	−	−	Wild type	−	80%+

### 3.5. Follow-up

The follow-up was conducted by telephone until August 2024. Case 1 had a follow-up period of 13 months and underwent total hysterectomy with bilateral salpingo-oophorectomy without any additional treatment post-surgery. Patients 2 and 3 died during the study. Patient 4 had a follow-up period of 23 months, had undergone radiotherapy and chemotherapy, had not undergone surgery, and had no signs of metastasis.

## 4. Discussion

### 4.1. Clinical features

Cervical neuroendocrine carcinoma accounts for only 1% to 3% of all cervical cancers.^[1]^ In the 5th edition of the WHO classification of female genital organ tumors in 2020, all neuroendocrine tumors occurring in the female reproductive system are discussed in a separate chapter, and neuroendocrine tumors are classified as well-differentiated neuroendocrine tumors or poorly differentiated neuroendocrine carcinomas.^[2]^ The former includes carcinoids and atypical carcinoids, while the latter includes SCNEC and large cell neuroendocrine carcinoma, with SCNEC being the most common. All 4 cases are SCNECs; the age of onset for SCNEC ranges from 14 to 78 years old^[3]^; and the ages of the 4 patients are 62, 51, 48, and 56 years, with an average age of 54.3 years. Although this type of tumor can secrete a variety of hormones and peptide neurotransmitters, the vast majority of patients with cervical neuroendocrine carcinoma do not exhibit clinical signs of hormonal abnormalities. However, a small number of cases may present with neuroendocrine symptoms such as carcinoid syndrome and Cushing’s syndrome.^[4]^ The clinical manifestations of this tumor are similar to those of other types of cervical cancer, with ~80% of its initial symptoms being vaginal bleeding.^[5]^ Out of the 4 patients, 3 presented with irregular vaginal bleeding.

### 4.2. Pathological features

Macroscopically, the mass may protrude as a polypoid growth or present as a cauliflower-like neoplasm, or the cervix may be abnormally shaped, resembling a “cylindrical” form. The cut surface was grayish-white, grayish-red, and nodular, with a texture that could be friable or hard, and the Hematoxylin-Eosin morphology of the tumor was similar to that of small cell carcinomas in other locations, such as the lung. The tumor cells were arranged in sheets, clusters, and cords. Tumor cells were oval or spindle-shaped, with significant atypia, indistinct cell borders, and a pronounced “crowding” phenomenon. The cell nuclei were oval, spindle-shaped, and oat-shaped, with deep staining and fine chromatin. The cytoplasm is scant, resulting in a high nuclear-to-cytoplasmic ratio. Nucleoli were not prominent, and nuclear mitoses were easily observed. Necrosis is visible in some areas and squamous and glandular epithelial differentiation may occur. Based on the histopathological morphology and immunophenotypic results, a definitive diagnosis can be made, which is an important auxiliary method for the differential diagnosis of cervical neuroendocrine carcinoma. It is generally believed that positivity for 1 epithelial marker (such as CEA/EMA/panCK) and positivity for at least 2 neuroendocrine markers (such as CD56/NSE/Syn/CgA) are definitive for diagnosis.^[6]^ Among neuroendocrine tumor markers, CD56 is the most sensitive but has poor specificity, CgA has higher specificity but lower sensitivity, and SYN has both good sensitivity and specificity. Therefore, the combined use of these 3 markers is of significant importance in the diagnosis of cervical small-cell neuroendocrine carcinoma.^[7]^ In 4 cases, 2 or more neuroendocrine markers were detected. The positivity rates were as follows: CD56, 100% (4/4); synaptophysin (SYN), 75% (4/4); and Chromogranin A (CgA), 50% (2/4). New neuroendocrine markers such as PGP9.5 and INSM1 have also been proven to have high positivity rates in cervical SCNEC, and even higher specificity and sensitivity than traditional markers (CD56, CgA, Syn).^[8,9]^ Cervical neuroendocrine carcinoma is associated with HPV infection, especially with a high prevalence of HPV16/18 infections, most of which show positive P16 staining.^[10]^ In 4 cases, P16 was diffusely and strongly expressed. The other marker panCK was positive, and the Ki-67 proliferation index was >70% in all cases.

### 4.3. Differential diagnosis

The differential diagnosis of uterine small-cell neuroendocrine carcinoma includes several considerations: first, low-grade squamous cell carcinoma or adenocarcinoma, poorly differentiated cancer with large cellular atypia, deeply stained nuclei, a high nuclear-to-cytoplasmic ratio, and morphological similarity to neuroendocrine carcinoma, especially in biopsy specimens where high vigilance is observed; immunohistochemistry can assist in differentiation; poorly differentiated squamous cell carcinoma or adenocarcinoma is positive for P63, P40, or CK18, whereas neuroendocrine markers are negative or focally positive. Second, metastatic neuroendocrine carcinoma: distinguishing metastatic small-cell neuroendocrine carcinoma from other types, particularly metastatic lung small-cell carcinoma, relies mainly on the clinical features and imaging findings to identify the primary site. At this time, TTF-1 cannot serve as a diagnostic marker for differential diagnosis, as both esophageal and cervical small cell carcinomas can be TTF-1 positive. Additionally, P53 in metastatic small cell lung carcinoma is often mutated, while P53 in cervical small cell carcinoma is typically wild-type.^[11]^ Furthermore, P16 is diffusely expressed in most cases of cervical small cell carcinoma, suggesting HPV association, which is not a characteristic feature of small cell lung carcinoma.^[12]^ In 4 cases, cases 1 and 2 were TTF-1 positive, but there were no space-occupying lesions in the lungs, and all 4 cases had wild-type P53 and diffuse positivity for P16. Third, endometrial stromal sarcoma: tumor cells are mostly short spindle-shaped or stellate, arranged in cords with cytoplasm that can be partially clear, and the tumor cells are positive for CD10 on immunohistochemistry. Fourth, cervical non-Hodgkin lymphoma: primary lymphoma of the cervix is very rare, with tumor cells diffusely distributed in sheets, lacking cohesion between tumor cells, and exhibiting significant atypia. Sometimes, the phenomenon of cell “squeezing” is also evident. Immunohistochemistry was positive for LCA, CD20, and CD3 but negative for neuroendocrine markers. Limited tissue sampling in biopsies means that the cellular morphology observed under a microscope is also limited. It is necessary to differentiate these cells from the lymphocytes in the cervical stroma. When tumor cells are scarce and the cell “squeezing” phenomenon is prominent, there is a risk of misdiagnosing them as mature lymphocytes, leading to a missed diagnosis. Therefore, it is crucial to be vigilant in these 4 cases, as the biopsy specimen from case 1 had a low number of tumor cells that were mistakenly identified as mature lymphocytes, resulting in a missed diagnosis.

### 4.4. Treatment and prognosis

Cervical small-cell neuroendocrine carcinoma (SCNEC) is extremely rare and there is currently no standardized treatment protocol. For early stage tumor lesions, the usual treatment involves total hysterectomy, bilateral salpingo-oophorectomy, and pelvic lymph node dissection followed by adjuvant radiotherapy and chemotherapy. For advanced-stage patients, a combination of radiotherapy and chemotherapy or a systemic chemotherapy regimen is recommended.^[13]^ Among the 4 patients, 2 died within 2 years after surgery, while the remaining 2 patients underwent combined treatments including radiotherapy and chemotherapy, and no recurrence has been observed to date. Compared with squamous cell carcinoma and adenocarcinoma of the cervix, cervical SCNEC is prone to lymph node metastasis and vascular invasion at an early stage, has a high recurrence rate, and has a poor prognosis.^[13]^ Studies have shown that the 5-year survival rate for early stage patients is 31.6% to 36.4%, while for late-stage patients, it is only 0% to 14%.^[14]^ According to the analysis of prognostic outcomes, tumor size, depth of tumor invasion, involvement outside the uterus, presence of lymph node metastasis, and International Federation of Gynecology and Obstetrics staging were factors affecting patient prognosis. Among these, lymph node metastasis and International Federation of Gynecology and Obstetrics staging are independent prognostic factors, which is generally consistent with the prognostic factors related to cervical neuroendocrine carcinoma reported in the literature.^[15]^

In conclusion, cervical SCNEC is an extremely rare and highly malignant tumor that progresses rapidly, with most cases being prone to metastasis at an early stage. Clinicians and pathologists should be familiar with the clinical and pathological characteristics of the tumor as well as treatment options. An accurate pathological diagnosis is important for patient prognosis and treatment.

## Acknowledgments

We thank the staff of the Department of Pathology at both participating hospitals for their assistance in specimen processing and slide preparation, and the nurses for their help in patient follow-up.

## Author contributions

**Conceptualization:** Pu Jin, Congyang Gu.

**Data curation:** Pu Jin, Congyang Gu.

**Formal analysis:** Congyang Gu.

**Funding acquisition:** Pu Jin, Xiaohua Liu.

**Investigation:** Pu Jin, Congyang Gu.

**Methodology:** Congyang Gu.

**Project administration:** Xiaohua Liu.

**Software:** Shiyue Huang.

**Validation:** Shiyue Huang.

**Visualization:** Shiyue Huang.

**Writing – original draft:** Pu Jin.
